# Nature’s contribution to poverty alleviation, human wellbeing and the SDGs

**DOI:** 10.1038/s41597-024-02967-0

**Published:** 2024-02-22

**Authors:** Mahesh Poudyal, Franziska Kraft, Geoff Wells, Anamika Das, Suman Attiwilli, Kate Schreckenberg, Sharachchandra Lele, Tim Daw, Carlos Torres-Vitolas, Siddappa Setty, Helen Adams, Sate Ahmad, Casey Ryan, Janet Fisher, Brian Robinson, Julia P. G. Jones, Katherine Homewood, Jevgeniy Bluwstein, Aidan Keane, Celia Macamo, Lilian Mwihaki Mugi

**Affiliations:** 1 Durrell Institute of Conservation and Ecology, School of Anthropology and Conservation, University of Kent, Canterbury, UK; 2 Department of Geography, Ludwig-Maximilians-Universität München, Munich, Germany; 3 School of Geosciences, University of Edinburgh, Edinburgh, UK; 4 Ashoka Trust for Research in Ecology and the Environment (ATREE), Bangalore, India; 5 Department of Geography, King’s College London, London, UK; 6 Stockholm Resilience Centre, Stockholm University, Stockholm, Sweden; 7 SCI Foundation, London, UK; 8 Department of Botany, Trinity College Dublin, Dublin, Ireland; 9 Department of Geography, McGill University, Montreal, Canada; 10 School of Natural Sciences, Bangor University, Bangor, UK; 11 Department of Anthropology, University College London, London, UK; 12 Institute of Social Anthropology, University of Bern, Bern, Switzerland; 13 Eduardo Mondlane University, Maputo, Mozambique; 14 School of Applied Sciences, Edinburgh Napier University, Edinburgh, UK

**Keywords:** Sustainability, Socioeconomic scenarios

## Abstract

Millions of households globally rely on uncultivated ecosystems for their livelihoods. However, much of the understanding about the broader contribution of uncultivated ecosystems to human wellbeing is still based on a series of small-scale studies due to limited availability of large-scale datasets. We pooled together 11 comparable datasets comprising 232 settlements and 10,971 households in ten low-and middle-income countries, representing forest, savanna and coastal ecosystems to analyse how uncultivated nature contributes to multi-dimensional wellbeing and how benefits from nature are distributed between households. The resulting dataset integrates secondary data on rural livelihoods, multidimensional human wellbeing, household demographics, resource tenure and social-ecological context, primarily drawing on nine existing household surveys and their associated contextual information together with selected variables, such as travel time to cities, population density, local area GDP and land use and land cover from existing global datasets. This integrated dataset has been archived with ReShare (UK Data Service) and will be useful for further analyses on nature-wellbeing relationships on its own or in combination with similar datasets.

## Background & Summary

Climate change and biodiversity loss are inextricably linked, sharing common drivers in human activities, and threatening human wellbeing and the possibility of achieving the Global Goals of Agenda 2030^1^. For example, climate- and biodiversity-related Sustainable Development Goals (SDGs) affect most of the other SDGs and are affected by a number of them – hence understanding synergies and trade-offs between the SDGs becomes crucial in attaining these goals overall^2^. However global policies such as those addressing biodiversity loss (e.g. the Strategic Plan for Biodiversity 2011–2020), climate change (Paris Agreement 2015), and sustainable development (Agenda 2030) pay insufficient attention to these linkages and trade-offs. There are considerable debates about how best to tackle climate change and biodiversity loss, often without due consideration to human wellbeing. Calls such as the ‘Half-Earth’ – reserving half of the planet for nature conservation^3–5^, or protecting 30% of the planet by 2030 fail to consider the potential costs to millions of people dependent on nature for their livelihoods^6,7^ or the fact that indigenous and forest-dependent local communities already protect and sustainably manage millions of hectares of forests worldwide often with greater success than traditional protected areas^8,9^.

Within this context, understanding the broader contribution of uncultivated (wild or less disturbed) ecosystems to the wellbeing of communities that use them directly remains relevant. A vast literature based on small sample studies exists exploring resource dependency^10–12^; natural resource management and governance^13–17^; gender^18–20^; markets^21–23^ among others. However, systematic pooled data analyses across multiple sites and countries are harder to find. Of the few exceptions, Centre for International Forestry Research (CIFOR)’s NTFP case comparison project focused specifically on non-timber forest products, livelihoods and conservation using a pool of 61 cases across Africa, Asia and Latin America^24^; Angelsen *et al*.^25^ used the resulting database covering 24 tropical countries for a global comparative analysis of environmental income and rural livelihoods; and Jagger *et al*.^26^ used the same database alongside RRI’s global forest tenure data to analyse tenure and forest income globally.

The Ecosystem Services for Poverty Alleviation (ESPA) Programme provided a common framework for several ambitious interdisciplinary projects to investigate the relationship between ecosystems and multidimensional wellbeing and/or poverty in countries throughout the global south between 2013 and 2019^27^. Building on these studies and supplementing them with comparable datasets from other research programmes, we have drawn on 11 datasets comprising 232 settlements and 10,971 households in ten low- and middle-income countries (Table 1), representing forest, savanna and coastal ecosystems, to analyse (i) how uncultivated nature contributes to multi-dimensional wellbeing, (ii) how benefits are distributed between households, and (iii) how governance (in the form of tenure regimes) mediates the relationship between uncultivated nature and wellbeing. These 11 datasets were selected for two key reasons: (1) they all collected similar data on ecosystem services and multidimensional human wellbeing using comparable surveys; (2) they all collected data within the same time period (2011–2015) making it easier to produce comparable estimates of wellbeing indicators.

**Table 1:** Overview of the original data included in the dataset.

Project acronym^(citation to original data/literature)^	Countries	No. of settlements	No. of households	Landscape types
*ACES*^30–33^	Mozambique	27	1614	Woodland, agriculture
*ASSETS*^34–38^	Colombia	11	195	Forest, agriculture, riverine
	Peru	9	250	Forest, agriculture, riverine
	Malawi	6	675	Forest, agriculture
*DELTAS*^39–41^	Bangladesh	63	1586	Coastal, marine, mangroves, agriculture
*P4GES*^42–44^	Madagascar	7	603	Forest, agriculture
*PEFESPA*^45^	India (Odisha)	4	127	Forest, agriculture
*PIMA*^46,47^	Tanzania	42	1922	North: Grassland pasture, savannah (arid/semi-arid) South: Miombo woodland (subhumid, significant woodland canopy throughout)
*SENTINEL*^48,49^	India	40	1112	Forest, agriculture
*SPACES*^50–55^	Kenya	4	786	Coastal, marine, mangroves, agriculture
	Mozambique	4	351	
*Miyun*^56^	China	15	1750	Forest, agriculture

This dataset was compiled for analyses in the research project ‘Nature’s contribution to poverty alleviation, human wellbeing and the SDGs’ (Nature4SDGs) (NERC Grant NE/S012850/1) and is now publicly archived with ReShare (UK Data Service)^28^. The dataset integrates secondary data on rural livelihoods, multi-dimensional human wellbeing, household demographics, resource tenure, and social-ecological context, thus focusing particularly on SDGs 1 (no poverty), 2 (zero hunger), 10 (reduced inequalities) and 15 (life on land). It primarily draws upon nine existing household surveys, and their associated site descriptions and qualitative interviews which informed and contextualised the surveys. It also draws upon existing global datasets on travel time to cities, population density, local area GDP, land use and land cover. Using this dataset, the Nature4SDGs project is specifically examining multidimensional wellbeing from the use of uncultivated nature; the role of common pool uncultivated resources in reducing income inequalities; and the consumption of wild protein across different social-ecological contexts. This dataset will potentially allow further analyses on nature-wellbeing relationships in future on its own or in combination with similar datasets.

## Methods

**Fig. 1:**
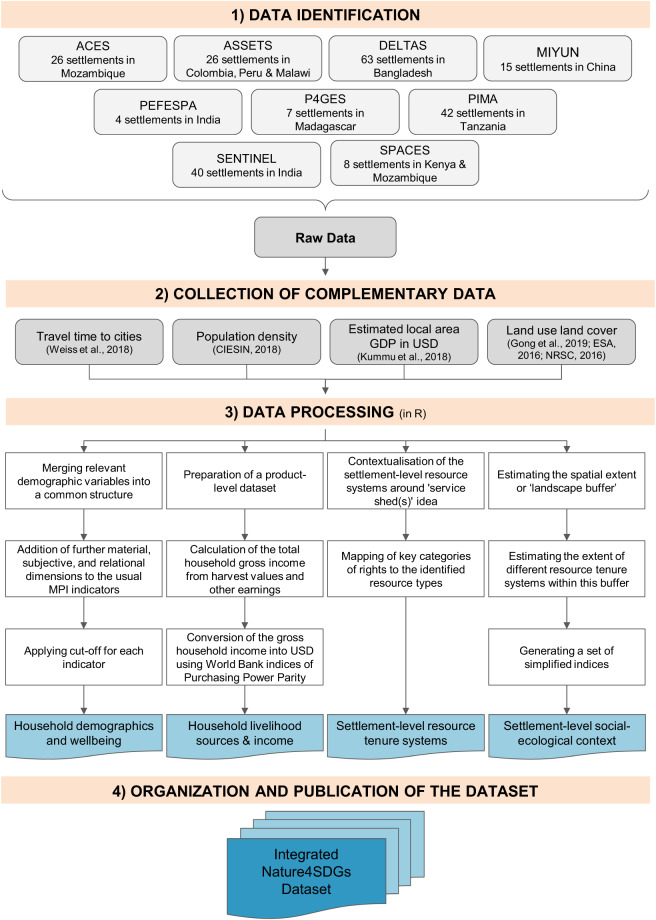
Summary of the steps and methods of creating the dataset.

The integrated dataset was developed in four main steps: (1) data identification; (2) collection of complementary data; (3) data processing; and (4) organization and publication of the dataset (Fig. 1).

### Data identification

**Fig. 2:**
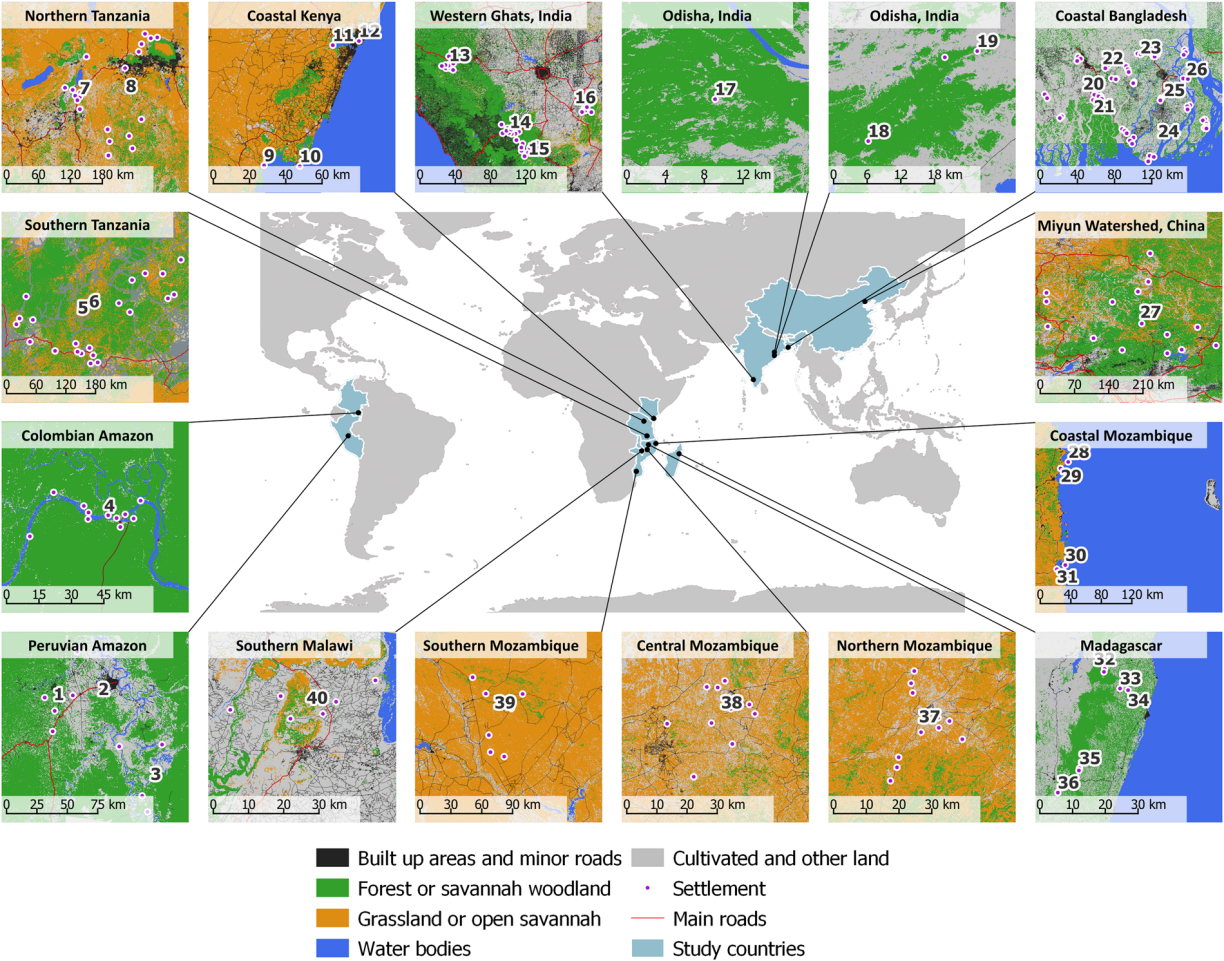
Locations of original sites of data collection included in this dataset.

The original household surveys were conducted during nine different research projects (see Table 1 and Figs. 1, 2); seven of those funded by the Ecosystem Services for Poverty Alleviation (ESPA) programme (https://www.espa.ac.uk), which ran from 2009 to 2018^29^. Each project had a different research focus, but all collected similar data on ecosystem services, livelihoods, and human wellbeing. All surveys collected information on livelihoods for a period between 2011 to 2015 and generated a variety of qualitative information from site descriptions and interviews. Owing to different sampling strategies in original surveys, while the sample of households is representative of each settlement, special attention needs to be paid when thinking about the population of settlements that this integrated dataset (or subsets of it) may represent. Full details of sampling strategies are available in the documentation of the original datasets^30–56^.

### Collection of complementary data

To supplement the household surveys, we also collated several global datasets to generate information on the social-ecological context of each settlement as close to the time period covering original data collection as possible (i.e. 2011–2015), including the travel time to cities^57^, population density^58^, estimated local area GDP in USD^59^, and land use land cover information^60–62^. This information was generated using data on the spatial location of the settlements, which is confidential and not included in this integrated dataset.

### Data processing

**Fig. 3:**
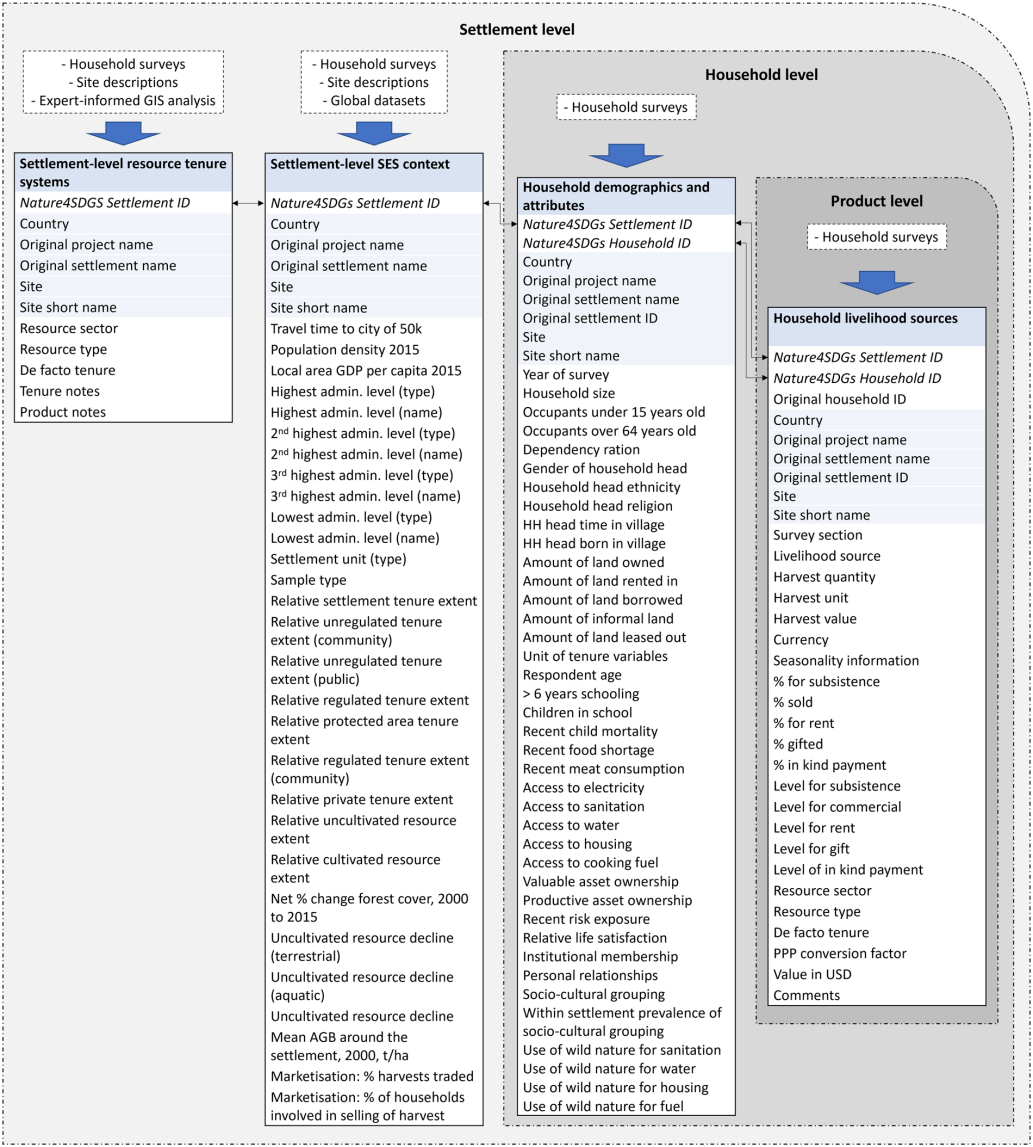
Summary of dataset structure and sources. Each column represents one of the archived csv files, and all variables are described fully in archived data documentation files^28^.

The dataset consists of four main components: Settlement-level resource tenure systems, settlement-level social-ecological context, household demographics and wellbeing, and household livelihood sources (Fig. 3). Below follows a description of the methods used to generate each component.

#### Settlement-level resource tenure systems

Our approach to identifying and mapping resource tenure prevalent at the site level followed existing frameworks on social-ecological systems^63,64^, tenure, and property rights^65–67^, together with the site/project-specific information on the types of resources available to the households and the *de facto* and *de jure* rights to those resources. We contextualised the settlement-level resource systems around the ‘service shed(s)’ idea^68^ and mapped key categories of rights to the identified resource types, focusing specifically on accurately mapping rights in practice (*de facto*) at all settlements based on the available qualitative data (participatory maps, land use change, resource trends) and household survey information (land holdings, access to resources).

This component of the dataset includes the variables resource sector, resource type, and *de facto* tenure. Sector is a higher-level classification of resources, similar to ‘RS1 Sector’ in Ostrom^63^, and includes categories such as agriculture, forest (natural), silviculture, fisheries and built environment. Each sector can include multiple resource types, which relate to primary use or purpose or some defining characteristics of the resource, including limitations. For example, farmland, agroforestry plots and home-gardens all fall under the ‘agriculture’ sector. Each resource type could be characterised as either ‘cultivated’ or ‘uncultivated’. The term tenure encompasses not just property rights but also wider institutions (such as who makes constitutional rules, who makes operational rules) within which resource use is embedded. While defining higher-level tenure types of resources, we limited ourselves to property rights. All common-pool resources comprise a class of goods that shares two attributes of importance for economic activities: (1) it is costly to exclude individuals from using the good either through physical barriers or legal instruments, and (2) benefits consumed by one individual subtract from the benefits available to others^69^. Schlager and Ostrom^65^ identify five property rights that are most relevant for use of common-pool resources: “access, withdrawal, management, exclusion, and alienation”^65^. For our resource tenure mapping exercise, particularly for uncultivated common pool resources, we are only looking at *de facto* access and withdrawal rights from those resource types. At the higher level, we have defined four resource tenure categories based on *de facto* rights to the resource type defined earlier. Higher-level tenure classification broadly corresponds to: (1) Privately owned resources (primarily cultivated); (2) Community-managed resources (sub-categorised into regulated or unregulated at the community level); (3) Protected area (sub-categorised into strictly protected, regulated access or *de facto* open access); and (4) Open access (unregulated public access).

#### Settlement-level social-ecological context

The variables included in this component of the dataset aim to describe the broader social and ecological context at the settlement level. The social variables are focused on the degree of market access in the village and population density. These variables were generated either directly from the surveys, or from existing spatiotemporal global datasets on travel time to cities^57^, population density^58^ and estimated local area GDP in USD^59^. The ecological variables are focused on characterising the land use land cover^60–62^ around each settlement, the associated *de facto* tenure regimes, as well as the relative extent of ‘wild’ or ‘uncultivated’ areas. All ecological variables were generated from existing spatiotemporal datasets.

We generated the variables for each settlement according to the following steps. Following established travel time-based methods for estimating elements of social-ecological landscape structure^70^, we first used information from local site descriptions and interviews to estimate the typical distance of one day’s return travel to a village in each site, based on common transport options and local geography. We then used this to calculate the diameter of a circular ‘landscape buffer’ within which to summarise settlement-level variables (varying from 3 km to 10 km in diameter for each village; see the methods summary accompanying the dataset for details). Next, we used land use land cover products and expert knowledge to generate estimates of the extent of different resource tenure systems within this buffer (see previous subsection for details on resource tenure system classifications), including the extent of uncultivated land covers. For example, within the landscape buffer around a village, the relative extent of different tenure classes within a landscape buffer could comprise 70% private land, 10% community regulated land and 20% protected area land with regulated access. In this example, if the community regulated and protected area were also uncultivated, the total uncultivated land within the buffer would be 30%. While the use of circular buffers are only approximate representations of actual resource catchment boundaries, our assumption is that the landscape metrics generated within these boundaries are correlated to some degree with characteristics of the underlying resource catchments.

#### Household demographics and wellbeing

This component of the dataset was generated in two parts; one on household demographics and one on multidimensional human wellbeing. For the part on *household demographics*, we used the household surveys to generate household-level variables on age, labour profile, socio-cultural grouping, and land ownership. Variables on age (dependency) and labour profile will affect which livelihood strategies a household can engage in, and the (per capita) benefit from these livelihoods within the household. Social capital and grouping variables are indicators of social difference that may indicate something about the way that they can interact with resource governance and other institutions. Variables on land ownership and tenure type provide household-level information on tenure, to complement the settlement-level ‘Resource Tenure System’ dataset. All variables were generated by importing each of the original household surveys into R^71^, then pulling relevant variables together into a common structure (mainly using the dplyr^72^ package). However, not all variables were available across all sites because they were not collected in some of the original datasets. The technical validation section describes how we dealt with missingness and data equivalence.

For the second part on *household multidimensional wellbeing*, we used the household surveys to generate a series of (mainly) binary household-level variables on different dimensions of human wellbeing. In doing so we sought to balance the need for detailed and locally contextualised measures with the need for cross-site comparability. Broadly, we defined wellbeing as having three dimensions (material, subjective and relational)^73^, and framed our measurement approaches based on the associated environment and development literature^50^, including subjective wellbeing^74^, human needs^75^, wellbeing in developing countries^76^, capabilities^77^, and relational wellbeing^78,79^. Definitions of these concepts vary^80^. As a starting point, Coulthard *et al*.^81^ provide a useful summary of HWB as comprising: “[…] a material dimension that emphasizes the objective resources a person has access to; a relational dimension that considers how social relationships influence what people can (or cannot) do; and a subjective dimension that takes into account a person’s level of satisfaction with the quality of life they achieve” (p. 299). Within this framing ‘basic needs’ approaches aim to understand if people are deprived in different subdimensions of material, subjective and relational wellbeing (e.g. health, education, shelter, life satisfaction, social relations etc.)^50^.

To develop standardised measures for these different subdimensions, we adapted the methods used to generate the Oxford Poverty & Human Development Initiative’s (OPHI) multidimensional poverty indicator (MPI)^82^. The MPI approach is grounded in Amartya Sen’s capabilities approach^77^ and is based on the ‘counting’ of different basic needs (or deprivations) that are met (or unmet) within a household^83,84^. It is already widely used to combine diverse data on wellbeing from different surveys, sites and countries^33,85,86^. Typically the MPI is generated in two steps. First, for each indicator of a basic need (e.g. years of schooling) a cut-off is applied (e.g. <6 years), below which a household is deprived for that dimension (i.e. it transforms each observed indicator into a deprived/not deprived binary variable). This cutoff value can differ between datasets depending on locally contextualised cutoffs and serves as a method of cross-dataset standardisation. In the second step, these binary indicators are added together to form a (weighted) multi-level ordinal index of relative aggregate deprivation across all households. A further cutoff is then applied to this variable to determine if a household is ‘poor’ (e.g. at least 1/3 of basic needs not met).

In generating this dataset, we used the MPI approach as a starting point and made two adaptations. First, in order to maintain the richness of our wellbeing data, we only implemented the first step to provide a multivariate set of binary variables of deprivation in different basic needs. These could be combined into a weighted ordinal variable or a binary MPI if needed. Second, in addition to the material basic needs usually measured in the MPI, we added further material dimensions (protein consumption, productive assets), as well as subjective (life satisfaction) and relational dimensions (institutions, autonomy)^74,87^. Most variables from the original surveys were transformed into binary indicators according to common thresholds in the latest MPI^88^ and literature associated with the original datasets^33,50,89,90^. The only exception is the life satisfaction variable, which was sufficiently similar between datasets that we could transform it into a four-level ordinal variable, and in doing so preserve more information on this dimension. All variables were generated in R using the dplyr package. A lower score means lower wellbeing for that dimension. Again, not all variables are available across all sites because they were not collected in some of the original datasets. We did not include a poverty line or wealth rank. These can be generated from the income information in the ‘Household Livelihoods’ dataset, and/or from the assets information in the original surveys.

#### Household livelihood sources

Broadly, the concept of livelihood includes economic as well as non-economic attributes of making a living. Apart from income, it covers the “social relationships and institutions that mediate people’s access to different assets and income streams” (p. 291)^91^. The second component of the dataset focuses on the economic aspect of livelihoods, i.e. income of households. To estimate household income, we prepared a dataset containing two types of livelihoods: harvests from cultivated (e.g. farms, aquaculture) and uncultivated (e.g. forests, fisheries) sources; and cash income from employment, businesses etc. For harvests, we generated information on the annual quantity and value of harvest of each product collected by each household and shares of harvests used for subsistence and sale. Cash income includes each household’s earnings from non-farm businesses and other sources, wage income and remittances. To derive the value of the harvest, we used the market price of each product, including for harvests that were not traded (e.g. subsistence harvests). To tackle the problem of missing price, we assigned the median price of a product calculated at the village level. Aggregation of harvest values and other earnings give the total household gross income. We then converted the gross household income into USD using World Bank indices of Purchasing Power Parity (PPP) in the year of the survey^92^.

Complete livelihoods were available for all original datasets except for PIMA (Tanzania) and PEFESPA (India). The PIMA survey gives the share of the harvest, instead of absolute quantities. Therefore, we are not able to estimate the gross value of harvest, only the proportion. The livelihood data of PEFESPA is incomplete in the sense that it has data on income from forest ecosystems but does not have data on income from the non-farm sector, wage income and remittances.

### Organisation and publication of the dataset

The integrated dataset is comprised of four components: (1) Household demographics and wellbeing, where each row is a household; (2) Household livelihood sources, where each row is a livelihood source associated with a particular household; (3) Settlement-level resource tenure systems, where each row is a natural resource associated with a settlement; (4) Settlement-level social-ecological context, where each row is a settlement. The four components can be linked together through corresponding ID variables (in a relational database structure). Figure 3 summarises the variables, ID links, and source data of each of the components. Some variables are not present across all sites. The implications of this missingness are discussed below in Technical Validation Section.

### Ethical approval

The integrated dataset described in this paper was produced under the Nature4SDGs project with full approval from the King’s College London’s Research Ethics Office on the ‘Use of Secondary Data in Nature4SDGs Project’ (KCL Ethics Ref: LRS-19/20-14886). As a general principle, all household level identifying information were already removed from the original datasets, as well as other village-level identifying information in some cases. All spatial data are kept confidential as per the original research ethics approval in the contributing datasets.

## Data Records

The integrated dataset is available through the ReShare repository^28^ and is comprised of four components, compiled as four separate .csv files: (1) Household demographics and wellbeing (**n4s_hh.csv**), where each row is a household; (2) Household livelihood sources (**n4s_lvl.csv**), where each row is a livelihood source associated with a particular household; (3) Settlement-level resource tenure systems (**n4s_rts.csv**), where each row is a natural resource associated with a settlement; and (4) Settlement-level social-ecological context (**n4s_setts.csv**), where each row is a settlement.

The four components can be linked together through corresponding ID variables (in a relational database structure). We also provide a further three csv files to help navigate and use the dataset: (1) **n4s_ids.csv**: to help link between csv files, a file with all corresponding IDs, including the IDs from the original source datasets (should you want to link back to the original source databases); (2) **n4s_variable_names.csv**: a csv file with all variable names and descriptions; and (3) **n4s_ls.csv**: which contains more detailed information on the extent, proportion and area of the different land covers and associated resource tenure systems in each settlement.

## Technical Validation

### Missingness, imputation, and data equivalence

While the original surveys shared a focus on ecosystem services and wellbeing, some of the variables differed slightly between the surveys. Additionally, each original survey has its own strengths and weaknesses in data quality (e.g. some have very robust livelihoods data, while others do not). In using this combined dataset, special consideration is therefore needed of missingness, as well as data quality and equivalence, between original datasets.

For data missingness, there are two types: (1) ‘Real’ missingness where, while a variable may have been collected in the original survey, there are occasionally a limited number of household values missing. This is due to more traditional issues around non-responses or enumerator errors in the original survey; and (2) ‘Question absence’ missingness, where the variable was not collected for an entire village or region in the original surveys.

Real missingness is coded as −9 in the dataset, while question absence is coded as −8.

In the published dataset, we have avoided imputing missing data for all variables except for harvest value (h_val) in the livelihoods data frame (n4s_lvl). For all other variables, missingness can be imputed from the raw data provided if users so wish. For harvest value, robust imputation requires site-specific knowledge on similarities between villages and harvest unit equivalence. We thus undertook to impute missing h_val values for the combined dataset.

Missingness of the harvest value data primarily occurred because respondents could not provide values for every harvest in every household (e.g. because some households do not trade every type of harvest). We thus used a hierarchical strategy to impute best estimates of harvest value where it was missing. In order of preference: (1) Where other households in the village had reported a harvest value for the same livelihood source (lvl_source) and unit (h_unit), we took the median of the harvest in the village; (2) Where the above was not possible, we used field notes on h_unit equivalences to impute the within-village median value of the same lvl_source with different units; (3) Where the above was not possible, we extended imputation to include median values from nearby villages that according to site experts were qualitatively similar in their socio-ecological context; and (4) Where the above was not possible, we used field notes and expert opinion from local site experts to estimate harvest values.

While we have sought to generate common variables across the original surveys, the varying origin of these variables means that data quality and equivalence need to be carefully considered when designing an analysis. In Section 4 of the data documentation^28^ we highlight particular issues with data quality and equivalence for each part of the integrated dataset. Generally, in any one analysis particular sites can be assumed to have more robust variables for particular constructs. Analyses can deal with this by either focusing only on sites with high quality variables, or by running multiple models (e.g. seeing if there are differences between one model with high quality sites only, and a second with all sites).

### Expert validation

During and after the generation of the integrated dataset, we validated variables for each site through an iterative process of data checking with one or more experts for each site who were involved in the original data collection. This included: generating and reviewing summary statistics of variables; reviewing apparent outliers and other anomalies; RTS and LULC map validation interviews, where we interactively viewed land cover maps and RTS for each settlement, reflected on their accuracy, and collaboratively linked RTS to specific land cover classes. This validation process was also facilitated through the testing of the dataset in four forthcoming analyses, during which remaining anomalies in the dataset were identified and checked. The validation process led to the exclusion of particular variables in a few settlements due to data quality issues in the original datasets, and to the refinement of RTS, livelihood and wellbeing classifications applied during our generation of the Nature4SDGs dataset.

## Usage Notes

We have done our utmost to ensure a robust standardised dataset. However, given the diversity and complexity of the original surveys, users need to be critical about data equivalence between sites and underlying biases in the sample of settlements. We thus strongly encourage users to consult the original documentation for each original dataset (referenced above) before using and drawing conclusions from the data for particular sites. We would also encourage users to consult the authors of this data paper on the use of data, who have a wealth of expert knowledge about the original datasets and specific knowledge of the sites. We highlight some specific usage notes below:

### Household demographics and wellbeing

- Some of the common variables on household demographics (e.g. health, education, assets, social capital) appear in a standardised binary form in the human wellbeing (HWB) part of the dataset. If needed, unstandardised ordinal, interval and/or continuous variables are available in the original datasets.
- Household-level tenure variables are focused on private land resources. Variables on common property terrestrial, aquatic and marine resources are at the settlement level and can be found in the settlement-level components of this dataset (‘Resource Tenure Systems’ and ‘Settlement-level SES context’). Private ownership in fisheries tends to be related to the ownership of a fishing vessel. This information is integrated into the ‘productive assets’ variable in the HWB dataset.
- We have included ethnicity and religion information as nominal variables. These variables can either be used as is, or can be further interpreted to group households in some more meaningful way for a particular analysis.
- Other useful information on household occupations (e.g. the presence of ‘elite’ occupations; employment in different sectors) and wealth ranks can be derived from the ‘Household Livelihoods’ data frame.
- We have not included variables on government aid, credit/savings (source and cost) or debt because this information was not widely available throughout the original surveys and the variables differ significantly where it was present. This information can be retrieved from the original datasets cited above if needed.

### Livelihoods

From the product level data set, we get the gross value of harvests i.e. the monetary value of harvest without deducting the cost of labour and other input costs. The reason for considering the gross value of harvest is that most of the original datasets do not have robust data on cost.

## Data Availability

The dataset was generated collaboratively by a team of researchers, working in the R or STATA platforms depending on their institutional and disciplinary preference. The final data generation code in R also draws upon a range of raw quantitative and qualitative datasets all with different owners, many of which contain confidential information that can be used to identify the names, demographics, opinions, and locations of individual respondents. Hence, not all code can be made publicly available. However, we have made all non-sensitive data generation code public through a public GitHub repository (https://github.com/mpoudyal/n4sdatagen), with clear explanation of those not in the public domain and how they fit within the data generation process in the README file within the repository. More importantly, we want to make clear that no custom code is necessary to utilise the collated dataset, and that the Data Description document on the data archive contains detailed written descriptions of all data generation processes including detailed description of variables developed in the process. Finally, parts of the codes containing sensitive information are available upon request to authors and after permission from the owners of the original datasets.
